# Role of an Intercostobrachial Nerve Block in Alleviating Tourniquet Pain: A Randomized Clinical Trial

**DOI:** 10.7759/cureus.22196

**Published:** 2022-02-14

**Authors:** Linda Le-Wendling, Barys Ihnatsenka, Anastasia Jones, Cameron R Smith, Erik Helander, Jeff Kedrowski, Olga C Nin, Amy M Gunnett, Yury Zasimovich

**Affiliations:** 1 Anesthesiology, University of Florida College of Medicine, Gainesville, USA

**Keywords:** nerve block, tourniquets, pain, intercostal nerves, brachial plexus block

## Abstract

Introduction

Tourniquet pain may have cutaneous and ischemic components. It is questionable whether blockade of a sensory nerve will help reduce ischemic pain. In addition, complete anesthesia of the axilla in the intercostobrachial nerve (ICBN) distribution is challenging to execute, and ICBN blockade has an inherently higher failure rate because of its variable anatomic location and source of innervation. We sought to determine the utility of an ICBN block for the prevention of tourniquet pain.

Methods

We conducted a single-center randomized controlled trial at a major academic medical center involving patients scheduled to undergo distal upper extremity surgery under ultrasound-guided supraclavicular brachial plexus block. Forty patients were randomized to receive an additional ICBN block or no ICBN block, with 22 allocated to the intervention and 18 to control. We collected data on the incidence of tourniquet pain and systemic anesthetic requirements.

Results

Initial contingency analysis examining the relationship between ICBN block placement and the development of pain using the two-tailed Fisher exact test failed to show that the presence or absence of ICBN block was associated with the development of tourniquet pain. χ^2 ^analysis failed to show that tourniquet time was significantly related to the development of tourniquet pain.

Conclusions

The overall incidence of tourniquet pain in the setting of a dense supraclavicular brachial plexus block for surgical anesthesia was low even without an ICBN block and even with tourniquet times greater than 90 min. Tourniquet pain was easily managed with small amounts of systemic analgesics.

## Introduction

Traditional teaching suggests that the intercostobrachial nerve (ICBN) should be blocked to prevent tourniquet pain. Data that support this teaching include older studies incorporating non-ultrasound-guided and distal approaches to the brachial plexus [1-4]. However, recent literature shows differences in opinion on the role of an ICBN block in preventing tourniquet pain [5-7].

From a mechanistic point of view, tourniquet pain described as a dull, pressured, and achy pain in the tissue underneath and distal to the tourniquet may have cutaneous and ischemic origins. However, if wrinkle-free padding is placed underneath the tourniquet to avoid skin folding and shear stresses to the skin, then the cutaneous component to tourniquet pain could be minimal. Additionally, blocking the ICBN, a cutaneous sensory nerve, should have minimal benefits in preventing tourniquet pain. 

Ischemic pain becomes progressively intolerable with the duration of ischemia and is much more difficult to blunt unless the regional anesthetic block results in dense anesthesia [8]. Indeed, some studies have demonstrated that denser blockade reduced the incidence of tourniquet pain [9,10]. An additional challenge to determining the contribution of the ICBN to tourniquet pain is the variability in success rates in anesthetizing the posteromedial upper arm [11].

Our primary aim was to determine the utility of an ICBN block for the prevention of tourniquet pain. We randomized patients who were scheduled to undergo distal upper extremity surgery under an ultrasound-guided supraclavicular brachial plexus block to receive an ICBN block or a control group that did not receive the block.

## Materials and methods

This single-center, prospective, randomized, single-blinded clinical trial was approved by the University of Florida Institutional Review Board (IRB201802525) and registered as a clinical trial (clinicaltrials.gov; NCT03797924; date of registration: 1/1/19; dates of patient enrollment: 4/25/2019 to 12/8/2020). Patients were included if they were 18 to 80 years old, American Society of Anesthesiologists status I to III, scheduled to undergo surgery of the upper extremity distal to the elbow with anticipated tourniquet use, and desiring regional anesthesia as the primary anesthetic. Patients were excluded if there was a contraindication to regional anesthesia, if they had primary block failure, or if they desired deep intraoperative sedation. As this is a pilot study, a sample size of 40 was chosen based on the potential availability of subjects and study completion within a period of 1 to 2 years.

After obtaining written informed consent, patients were allocated via a computer-generated randomization chart to receive an additional ICBN block or no ICBN block. The intraoperative team was blinded to the presence or absence of ICBN block by the application of a wide preparation with tinted chlorhexidine over the axilla and anterior chest wall. Patients received midazolam for the block procedure and had a minimal recall of the presence of an additional nerve block.

Brachial plexus blocks were performed at the supraclavicular fossa using 20 to 30 mL of 0.5% ropivacaine under ultrasound guidance. The ICBN block was performed with 10 to 20 mL of 0.5% ropivacaine in the plane deep to the pectoralis minor and/or serratus anterior muscle over the second and third intercostal space. If the patient was randomized to the no ICBN block group, the anterior chest and axilla were prepared with tinted chlorhexidine, but no ICBN block was performed. Before proceeding to the operating room, patients were evaluated for the success of the primary brachial plexus block with a pinprick sensation and motor testing in the radial, ulnar, median, and musculocutaneous nerve distributions. The ICBN distribution was tested with a pinprick on the anteromedial and posteromedial aspect of the upper arm.

Tourniquet management

A straight pneumatic tourniquet (Stryker Instruments, MI, USA) was placed on the arm over soft padding, according to local protocol. Exsanguination with an Esmarch bandage was performed before tourniquet inflation to 250 mm Hg. The intraoperative anesthesia team documented the tourniquet inflation and deflation time.

Sedation, supplemental analgesia, and management of tourniquet pain

Patients were provided with midazolam (up to 2 mg) if requested and music via earphones for intraoperative anxiolysis. No opioids were administered before the incision. At the first patient complaint of pain, the intraoperative anesthesia team was asked to document the quality and location of pain, and 50 mcg of fentanyl was administered if the patient requested it. If pain persisted, the fentanyl dose could be repeated one additional time. If the pain persisted despite two doses of fentanyl, a propofol infusion was started at 50 mcg/kg/min and titrated at 10 mcg/kg/min every two to three minutes to achieve the lowest Richmond Agitation-Sedation Scale (RASS) score resulting in patient comfort.

Outcome parameters

The primary aim of the study was to determine if the ICBN block affected the incidence, time to onset (in minutes), and severity of tourniquet pain, defined by the presence of dull, aching pain underneath the tourniquet. Secondary endpoints of the study were ICBN block effect on intraoperative opioid consumption and depth of anesthesia to alleviate tourniquet pain.

Sample size

Because the incidence of tourniquet pain was not known before the commencement of the study, a retrospective power analysis was conducted. Having found that one of 25 participants in the ICBN block group developed pain and three of 25 in the non-blocked group (proportions of 0.04 and 0.12, respectively) and using a null difference in the proportions of 0.2, the study had 95.4% power to detect such a difference.

Statistical analysis

Data collected included patient demographic information (age, sex, ethnicity, height, weight, and body mass index), sensory/motor distributions of the musculocutaneous/radial/median/ulnar/ICBN blocks, time of tourniquet inflation and deflation, quality and location of intraoperative pain, use of intraoperative fentanyl or propofol, and RASS scores.

Data were analyzed using the JMP Pro 15 statistical software package (SAS Institute, Cary, NC, USA). Data were tested for normality. Initial contingency analysis was made by comparing the frequency of tourniquet pain in patients treated with the ICBN block and those not treated with the block. This was followed by comparing tourniquet inflation time in those who did and those who did not develop tourniquet pain with logistic fit models. First, the role of tourniquet inflation time was examined across all study participants. Subsequently, the role of tourniquet inflation time was examined in each of the treatment groups separately. Last, a mixed-models analysis of variance was undertaken in a model incorporating tourniquet time, treatment group, and the interaction between treatment group and tourniquet time. All data are presented as means ± SD for one-way analyses and parameter estimates and 95% confidence intervals for logistic fit analyses.

## Results

**Figure 1:**
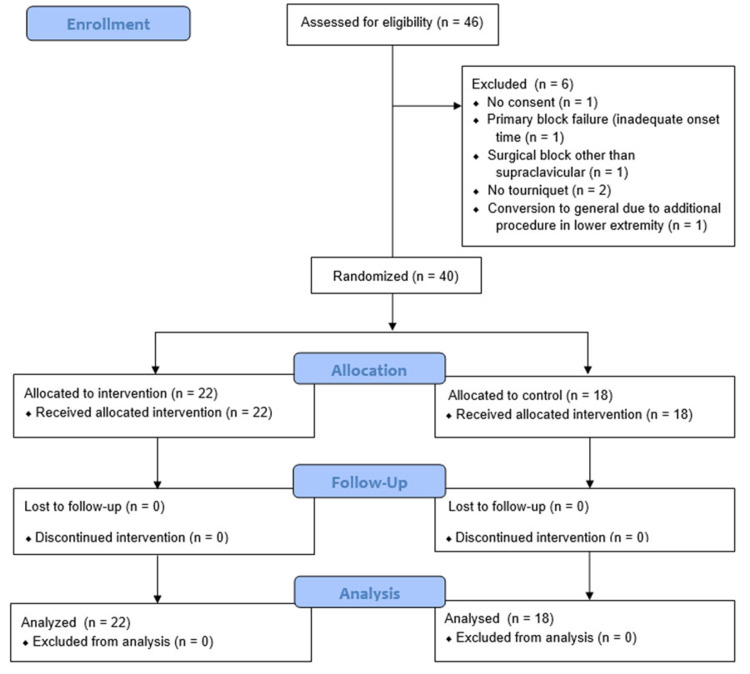
CONSORT flow diagram.

Forty-six patients were enrolled; after the exclusion of six patients for various reasons, 40 patients completed the study, and data were collected for analysis (Figure 1). Patient demographics are reported in Table 1. All patients received midazolam, and 13 patients received additional doses of alfentanil (up to 1000 mcg) for block performance. Block characteristics are reported in Table 2.

**Table 1:** Patient demographics. ASA: American Society of Anesthesiologists. BMI: body mass index. ORIF: open reduction and internal fixation. SD: standard deviation. TFCC: triangular fibrocartilage complex

Demographic Variables	Block Group	Control Group	P-Value
Age (years) (median)	61.5	56.5	0.05
Gender (n, %)			0.20
Male	9 (41%)	11 (61%)	
Female	13 (59%)	5 (39%)	
BMI (mean ± SD)	29 ± 6.4	28 ± 8	0.06
ASA status (n, %)			0.78
I	5 (23%)	5 (28%)	
II	6 (27%)	6 (33%)	
III	11 (11%)	7 (39%)	
Surgery duration (min) (mean ± SD)	80 ± 42	95 ± 63	0.40
Tourniquet time (min) (mean ± SD)	75 ± 34	84 ± 50	0.50
Tourniquet time (min) (range)	15–139	15–161	
Tourniquet time >90 (min) (n)	8	10	
Tourniquet time (min) (n)			
<30	3	3	
30–60	4	5	
60–90	6	0	
90–120	6	5	
>120	2	5	
Type of surgery (n)			
Distal radius ORIF	10	7	
Tendon transfer/reconstruction	4	3	
Hardware removal	4	1	
Right wrist arthroscopy, TFCC debridement, ulnar/radial osteotomy	2	1	
Phalangeal fracture ORIF	1	0	
Wrist reconstruction	1	5	
Suspensionplasty	0	1	

**Table 2:** Block characteristics. Data are reported as mean ± SD or n (%). ICBN: intercostobrachial nerve block

Characteristic	Block Group	Control Group	P-value
Block procedure length (min) (time to perform supraclavicular + ICBN block)	12 ± 4.01	8 ± 2.28	<0.001
Time from block to incision (min)	78 ± 52	91 ± 37.62	0.37
ICBN block failure	4 (18%)	N/A	N/A

Eighteen patients had tourniquet times of 90 min or longer. Four patients in the ICBN group failed to have sensory changes in the anteromedial and/or posteromedial aspect of their upper arm, representing an 18% incidence of failed ICBN blocks. None of these patients with failed ICBN blocks complained of tourniquet pain.

Four patients complained of tourniquet pain, but two declined analgesia for management (Table 3). The incidence of tourniquet pain was 5% in the ICBN block group and 15% in the no ICBN block group. Two of the subjects with tourniquet pain were managed each with two successive doses of fentanyl at 1:08 and 1:34 h and 1:35 and 1:56 into their tourniquet times. Propofol was not initiated for any of the patients. Of the patients with tourniquet pain, one was in the ICBN group, and three were in the group that did not receive an ICBN block (Table 4). The times from tourniquet inflation to complaints of tourniquet pain in these four patients were 0:49, 1:15, 1:32, and 1:53 h.

**Table 3:** Tourniquet pain outcomes. Data are reported as counts or mean ± SD. ICBN: intercostobrachial nerve block

Tourniquet Pain	Block Group	Control Group	P-Value
Present	1	3	0.20
Number of patients who reported tourniquet pain and had a failed ICBN block on preoperative screening)	0	N/A	
Onset time from incision to tourniquet pain (min)	75	85 ± 32.6 (49, 92, 113 min)	
Opioid consumption			
Number of fentanyl doses in 50-mcg increments	0	5	
Time to first fentanyl dose (min)	N/A	82 ± 19	

**Table 4:** Raw data for subjects with tourniquet times >90 min ICBN: intercostobrachial nerve block

ICBN Block	Presence of Tourniquet Pain	Tourniquet Time
Yes	No	1:30
Yes	No	1:31
Yes	Yes	1:36
Yes	No	1:37
Yes	No	1:44
Yes	No	1:49
Yes	No	1:53
Yes	No	2:02
Yes	No	2:04
Yes	No	2:19
Yes	No	2:30
No	No	1:31
No	No	1:50
No	No	1:51
No	No	1:56
No	Yes	2:03
No	Yes	2:23
No	No	2:41

Of the three patients who received intraoperative fentanyl, two complained of tourniquet pain. One patient in the no ICBN block group was given 50 mcg of fentanyl by the intraoperative anesthesia provider for anxiety.

**Figure 2:**
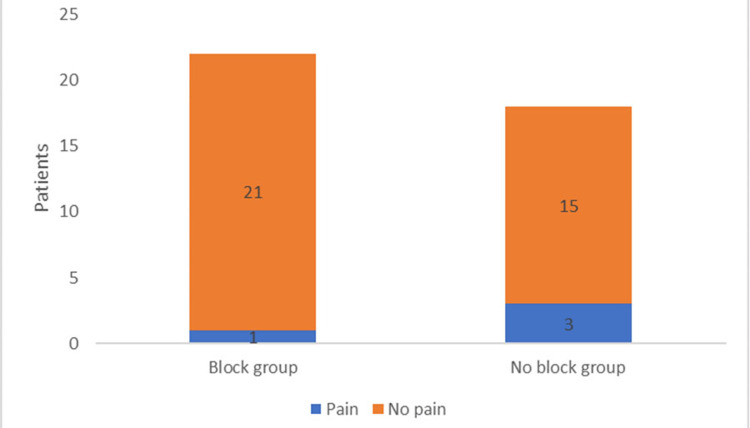
Development of pain between groups. Initial contingency analysis examining the relationship between intercostobrachial nerve (ICBN) block placement and the development of pain using the two-tailed Fisher exact test demonstrated that the presence or absence of the ICBN block was not significantly associated with the development of tourniquet pain (p = 0.31). When examined again using a one-tailed Fisher exact test to examine the probability that tourniquet pain was more likely in patients not receiving the ICBN block, the results remained statistically nonsignificant (p = 0.23).

Initial contingency analysis examining the relationship between ICBN block placement and the development of pain using a two-tailed Fisher exact test failed to show the significance that the presence or absence of ICBN block was associated with the development of tourniquet pain (p = 0.31). When examined again using a one-tailed Fisher exact test to examine the probability that tourniquet pain was more likely in patients not receiving the ICBN block, the results remained statistically nonsignificant (p = 0.23; Figure 2). When comparing patients who did and did not report tourniquet pain, tourniquet times were significantly longer in patients who did experience tourniquet pain (75.1 ± 41.5 vs. 118.8 ± 19.6 min, p = 0.0464). However, this difference was no longer significant when broken down into the treatment groups (ICBN vs. no ICBN groups). Tourniquet times were not significantly different in patients who did and did not develop tourniquet pain in the group that received the ICBN block (74.7 ± 35.8 vs. 96 min (no SD as n = 1), p = 0.5663) or in the group that did not receive the ICBN block (75.7 ± 49.8 vs. 126.3 ± 15.3 min, p = 0.1074).

Similarly, mixed-model analysis of variance was undertaken using a model that examined the effects of tourniquet times (entered as a numeric variable instead of a dichotomized variable), treatment group (ICBN vs. no ICBN), and the interaction between tourniquet times and treatment group. Neither tourniquet times (-0.02, 95% CI -0.07 to 0.01, p = 0.21) nor presence of the ICBN block (0.40, 95% CI -1.43 to 2.45, p = 0.61) was significantly associated with the development of tourniquet pain and there was no interaction effect between these two factors (p = 0.79, 95% CI -0.04 to 0.04).

## Discussion

In the setting of an effective ultrasound-guided supraclavicular block for surgical anesthesia, the incidence of tourniquet pain was low. Even when tourniquet times exceeded 1½ h, the incidence of tourniquet pain was low (approximately one in three patients without the ICBN block and one in five patients with the ICBN block). Furthermore, the number of systemic analgesics required to manage tourniquet pain when it manifested was minimal. Propofol infusion was not needed to manage tourniquet pain.

One of the reasons for such effective brachial plexus blocks is the use of moderate volumes (20-30 mL) of a higher concentration of local anesthetic, 0.5% ropivacaine. The reason a supraclavicular block was performed instead of a more distal approach was twofold. When compared to an axillary block, the supraclavicular block more reliably covers the axillary nerve and musculocutaneous nerve, as well as the medial cutaneous nerve of the arm and forearm. In addition, the placement of an infraclavicular block theoretically more likely spills into the area where the ICBN traverses and results in an unanticipated spread in this distribution [12]. In addition, recently published work by Wahal et al. [13] demonstrated that the instillation of a local anesthetic around the femoral artery in an attempt to block the afferent sympathetic fibers running with the vessel effectively reduced tourniquet-induced hypertension in patients undergoing total knee arthroplasty. If this phenomenon applies more broadly, then the close periarterial application of local anesthetic when performing a supraclavicular brachial plexus block may limit the perception of ischemic pain in the affected limb regardless of whether the ICBN is blocked.

It is notable that with tourniquet times exceeding two hours, the addition of an ICBN block to blunt tourniquet pain may be of some benefit. However, using a mixed-models logistic fit model, the ICBN block did not appear to reduce the likelihood of tourniquet pain. The effect of tourniquet time remained nonsignificant in terms of an effect on pain, although our sample size was too small to make definitive conclusions. Notably, the patient with the longest tourniquet time did not receive an ICBN block and did not complain of tourniquet pain. 

We used a chest wall approach to anesthetize the ICBN because we perceive that the reliability of anesthetizing this nerve is better, and the coverage is greater when targeted on the chest wall instead of in the axilla, where terminal branches may be missed. Numerous fascial plane block techniques have been proposed to anesthetize the lateral cutaneous branch of the T2 intercostal nerve, including PECS2, subpectoral intercostal plane, serratus anterior plane, and serratus-intercostal fascial plane blocks [11,14]. Because the T2 intercostal nerve traverses from between the internal and innermost intercostal muscles to the skin, we presume that local anesthetic spread on any of the planes between those two target planes at a proximate cephalad-caudad and anterior-posterior location should result in reliable coverage of the ICBN. Despite this more tailored approach, we still had an 18% failure rate for axillary coverage.

Samerchua et al. [11] published an anatomical cadaver study in which they delineated the contributions of the ICBN and noted that in all cases, there was a T2 contribution; however, up to 25% of ICBNs also receive contributions from T1 and T3. In addition, the ICBN was noted to have a higher degree of staining when the ICBN block was performed proximally at the level of the second and third rib near the anterior axillary line. On the other hand, only 43% of the axillary branches of the ICBN nerve were stained with a distal approach in the axillary fossa.

With the low reliability of success of an ICBN block, a low incidence of tourniquet pain in the setting of a successful brachial plexus block (even without anesthetizing the ICBN), and the ease of management of tourniquet pain with minimal systemic analgesics/sedatives, we do not believe that the addition of an ICBN block is warranted for the prevention of tourniquet pain, even with anticipated prolonged tourniquet times.

Limitations

We could not enroll patients in this study without the use of sedatives for block performance, given the surgical population at our institution. However, the time from block completion to initiation of surgery was as long as four hours; frequently, the sedative effects of the block procedure were minimal by that time.

## Conclusions

The overall incidence of tourniquet pain in the setting of an effectively dense supraclavicular brachial plexus block for surgical anesthesia was low, even without the addition of an ICBN block. This tourniquet pain can be easily managed with small increases in systemic analgesics.
